# Whole-Genome Resequencing to Evaluate Life History Variation in Anadromous Migration of *Oncorhynchus mykiss*

**DOI:** 10.3389/fgene.2022.795850

**Published:** 2022-03-15

**Authors:** Erin E. Collins, Nicolas Romero, Joseph S. Zendt, Shawn R. Narum

**Affiliations:** ^1^ Hagerman Genetics Laboratory, Columbia River Inter-Tribal Fish Commission, Hagerman, ID, United States; ^2^ Yakama Nation Fisheries, Yakima/Klickitat Fisheries Project, Klickitat, WA, United States

**Keywords:** *Oncorhynchus*, steelhead, anadromy, whole-genome resequencing, Klickitat River

## Abstract

Anadromous fish experience physiological modifications necessary to migrate between vastly different freshwater and marine environments, but some species such as *Oncorhynchus mykiss* demonstrate variation in life history strategies with some individuals remaining exclusively resident in freshwater, whereas others undergo anadromous migration. Because there is limited understanding of genes involved in this life history variation across populations of this species, we evaluated the genomic difference between known anadromous (*n* = 39) and resident (*n* = 78) *Oncorhynchus mykiss* collected from the Klickitat River, WA, USA, with whole-genome resequencing methods. Sequencing of these collections yielded 5.64 million single-nucleotide polymorphisms that were tested for significant differences between resident and anadromous groups along with previously identified candidate gene regions. Although a few regions of the genome were marginally significant, there was one region on chromosome Omy12 that provided the most consistent signal of association with anadromy near two annotated genes in the reference assembly: COP9 signalosome complex subunit 6 (CSN6) and NACHT, LRR, and PYD domain–containing protein 3 (NLRP3). Previously identified candidate genes for anadromy within the inversion region of chromosome Omy05 in coastal steelhead and rainbow trout were not informative for this population as shown in previous studies. Results indicate that the significant region on chromosome Omy12 may represent a minor effect gene for male anadromy and suggests that this life history variation in *Oncorhynchus mykiss* is more strongly driven by other mechanisms related to environmental rearing such as epigenetic modification, gene expression, and phenotypic plasticity. Further studies into regulatory mechanisms of this trait are needed to understand drivers of anadromy in populations of this protected species.

## 1 Introduction

Variation in life history traits is often advantageous to the persistence of natural species, and salmonids retain diverse life history types to contend with environmental stochasticity (e.g., Moore et al., 2014). Species such as *Oncorhynchus mykiss* demonstrate variation in migratory life history with some individuals remaining exclusively resident in freshwater, whereas others exhibit anadromous migration from freshwater rearing environments to the ocean before returning to natal areas to spawn. Populations can consist of primarily anadromous or resident fish, or there can be a mixture of resident and anadromous individuals within a single population. Anadromy is rare, <1% of all fish species are anadromous (McDowall 1988), because the biological cost includes challenges of surviving in both fresh and saltwater habitats (e.g., physiological changes to regulate ions, long distance migrations, and predation rates) and must be outweighed by benefits such as improved productivity and access to seasonally available resources to feed and spawn. Because of smoltification and migration of anadromous fish, reproduction is delayed for adults; however, they benefit from large size and high fecundity gained from highly productive marine feeding habitat. Ultimately, life history development is a complex trait and is influenced by genetics, environment, and individual fitness (Fleming and Reynolds 2004). Variation in the degree of anadromy or residency of *O. mykiss* populations impacts the abundance, diversity, resilience, structure, and productivity of *O. mykiss* populations (Waples et al., 2008). There are greater benefits for female *O. mykiss* to migrate and grow to a larger size before spawning compared to males. Larger female *O. mykiss* experience increased fecundity, increased egg size, improved redd site protection, can dig deeper into the substrate, and can attract more mates (Quinn et al., 2011). Whereas, male *O. mykiss* are more likely than females to be resident due to an option for males to spawn at a small size with the “sneaky/sneaker” male tactic (Fleming and Reynolds 2004), and this variation in males was the focus of this study.

*Oncorhynchus mykiss* are of immense economic, ecological, and cultural importance throughout their range. Populations of *O. mykiss* are considered partially migratory due to the presence of both anadromous, “steelhead,” and resident, “rainbow trout,” forms of the species. These life history types of *O. mykiss* are sympatric and can produce offspring of either life history type (Christie et al., 2011; Courter et al., 2013; Sloat and Reeves 2014). Anadromy of *O. mykiss* are initiated by a combination of environmental and genetic cues and the genetic component is currently not well understood across much of the geographic range of this species. A continued presence of both resident and anadromous *O. mykiss* is critical to population resilience to environmental or anthropogenic disturbances (Moore et al., 2014).

Previous studies have identified candidate genes or regions in the genome associated with the trait for anadromy in steelhead, but association has been limited to particular populations. For example, Pearse et al. (2019) and Martínez et al. (2011) detected a “migration supergene” in an inversion on chromosome Omy05, where the ancestral form is associated with anadromous steelhead and the derived form is associated with resident rainbow trout. The signal from the chromosome Omy05 inversion was significant in coastal populations from southern portions of the range but had low variability for other populations of steelhead (inland or northern latitudes). Studies of anadromy in specific populations of *O. mykiss* have identified putative candidate genes associated with resident versus migratory life history (e.g., Nichols et al., 2008; Narum et al., 2011; Hale et al., 2013), but these candidates have not been directly validated in further studies. In addition, previous studies (Nichols et al., 2008; Narum et al., 2011; Hale et al., 2013; Hecht et al., 2013) detected outlier loci of small effect throughout the genome for anadromy, indicating an extremely polygenic trait, and very few of the same loci were detected in multiple analyses and little overlap was observed between populations tested, indicating a lack of a conserved gene region or high environmental plasticity for the trait. Thus, studies to date indicate that the genetic basis of anadromy is controlled in a population specific manner. However, there is currently a paucity of data from many populations, limiting our understanding of the relative importance of shared alleles and population specific anadromy.

Steelhead in the Klickitat River, WA, USA, are geographically and genetically intermediate between coastal and inland genetic lineages (Blankenship et al., 2011; Collins et al., 2020). The geography along the Klickitat River and its tributaries is diverse and includes waterfalls that act as partial barriers to migration, leading to reproductive and genetic isolation for populations in some areas (Narum et al., 2006; Narum et al., 2008). Another genetic influence on Klickitat River *O. mykiss* populations has been the stocking of summer-run hatchery steelhead, known as Skamania steelhead, since 1961, and the Skamania steelhead may have overlapping spawning timing with natural origin summer steelhead, but with limited gene flow (Narum et al., 2006). Skamania steelhead were originally derived from steelhead from the Washougal and Klickitat Rivers and have been strongly selected for early spawning times (Crawford 1979). Resident *O. mykiss* occur throughout substantial portions of this drainage, especially in headwater tributaries with lower flows and water temperature than large tributaries or the mainstem Klickitat River (Narum et al., 2008; Evans et al., 2021). Because Klickitat River anadromous steelhead are listed as threatened and are protected under the Endangered Species Act, identifying that the genetic component of anadromy may be a useful conservation tool to assist with recovery of steelhead in this system.

In this study, we aimed to use whole-genome resequencing to assess underlying genetic mechanisms for the trait of male anadromy in *O. mykiss* from the Klickitat River. This study combined high marker density throughout the genome and phenotypic data from long-term tracking of individuals to test for regions of the genome associated with resident versus anadromous migration patterns. Further, markers from previously identified candidate genes were tested for differences among collections. A genetic sex marker on the basis of the sexually dimorphic on the Y chromosome region (sdY; Yano et al., 2013) was used to identify males and females, enabling analyses to focus on sex specific migration patterns in males to avoid sex bias when comparing anadromy life traits due to the biological constraint of rare resident female rainbow trout.

## 2 Materials and Methods

### 2.1 Sample Collection and Phenotypes

**FIGURE 1:**
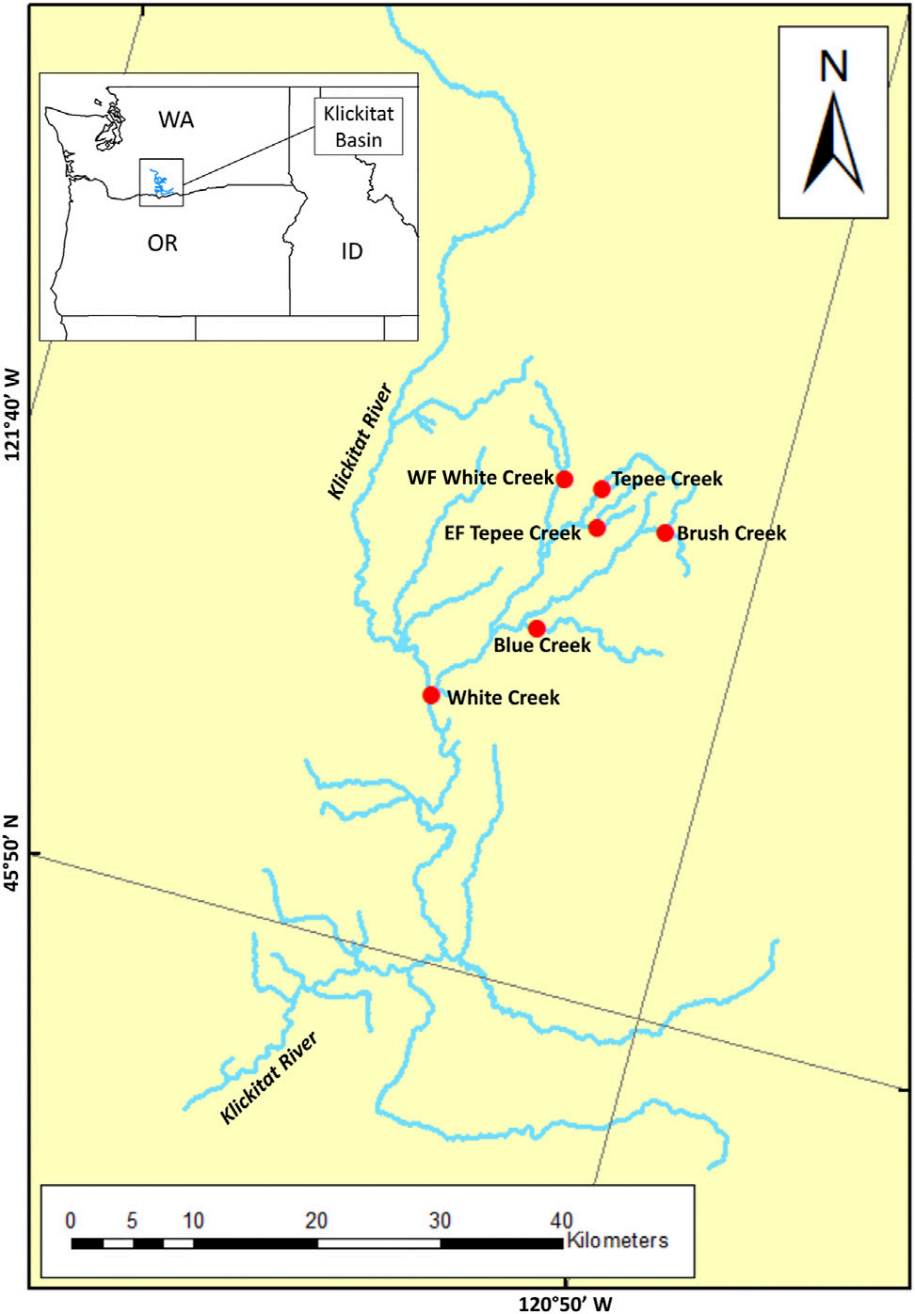
Map of sample sites within the White Creek tributary of the Klickitat River, WA, USA, marked with a red circles. Individual samples within this tributary were identified as either resident (*n* = 78) or anadromous (*n* = 39) on the basis of tracking migratory fish patterns with passive integrated transponder (PIT) tags.

Samples of juvenile *O. mykiss* were collected from the Klickitat River *via* electrofishing from the following tributaries of the White Creek drainage: White Creek, West Fork White Creek, Blue Creek, Brush Creek, Tepee Creek, and East Fork Tepee Creek (Supplementary Table S1 and Figure 1). All individual *O. mykiss* were captured as juveniles (parr appearance) in multiple sites of the White Creek drainage between 2009 and 2019 and were inserted with a 12.5-mm full-duplex passive integrated transponder (FDX-B PIT) tag (Biomark, Boise, ID, USA) to track individual migration patterns and a non-lethal fin clip was collected for genetic analyses. Fish were tracked for multiples years to determine migratory classification as either resident or anadromous for genomic analyses.

Sampling was ongoing and conducted every year with marked fish continuously monitored *via* passive mark recapture methods. Captured fish were initially scanned with a handheld PIT tag scanner to identify previously PIT-tagged fish (recaptures) from non-tagged fish at each sample site. A PIT tag was inserted in each non-tagged *O. mykiss* ≥ 65 mm. Sample date, PIT tag code, and biometric data were recorded from each fish. In addition, a non-lethal fin clip for genetic analysis was collected from first time captured fish. Each sampled fish was returned to the stream at their capture location. Movement patterns were tracked through a combination of active and passive mark-recapture methods. Electrofishing surveys were conducted at each site annually to actively monitor fish. Electrofishing was the method used to both capture fish for marking and to manually recapture previously tagged fish. We used electrofishing as a method to classify resident fish by establishing a temporal record of recapture events for a given individual.

A network of PIT tag interrogation arrays in the Klickitat River and Columbia River were used to passively monitor migratory behavior. PIT tag interrogation arrays located in tributaries to the Klickitat River were designed to interrogate the entire wetted channel width *via* channel spanning PIT tag arrays. The mainstem Klickitat River floating array interrogated approximately two-thirds of the mainstem wetted channel. Sites varied from having a minimum of two arrays to a maximum of three arrays. Depending on stream width, arrays consisted of a single antenna to up to three adjacent antennas. Arrays were configured by placing antenna arrays perpendicular to the stream channel. PIT tag arrays were spaced five to 10 m apart along the longitudinal stream axis. For each fish detection, the PIT tag code, date/time stamp, and unique antenna number were recorded to a data logger buffer. Directionality of movement and temporal movement patterns were subsequently determined from these data. Juvenile bypass interrogation arrays at Bonneville Dam operate continuously, except for a short non-operational period in the winter. In addition, an array is towed through the Columbia River estuary to detect PIT-tagged fish throughout the year. All PIT tag interrogation sites were operational during the juvenile out-migration period.

Individual O.mykiss samples were classified as either resident or anadromous based on multiple years of mark-recapture sampling and monitoring. Anadromous fish were identified as those that successfully outmigrated from the Klickitat River and were either last detected in the mainstem Columbia River presumably headed to the ocean or returning from the ocean. Fish were classified as freshwater residents if they remained in freshwater for a minimum of 2 years between initial and final recapture events and did not register at any of the downstream PIT tag arrays.

### 2.2 Molecular Methods

Prior to whole-genome resequencing, specimens were genotyped with a panel of single-nucleotide polymorphism (SNP) markers to identify sex of samples and to account for kinship and population structure. A Chelex 100 method (Sigma-Aldrich, St. Louis, MO) was followed to extract DNA from the tissue of a total of 198 *O. mykiss* individuals (Supplementary Table S1). The genotyping-in-thousands by sequencing method (GT-seq; Campbell et al., 2015) was employed for all samples. Samples were genotyped with a panel of 376 SNPs (Collins et al., 2020) that were a mix of putatively neutral and adaptive markers, a sex marker, and markers that identify closely related species of cutthroat trout (*O. clarkii*). Briefly, our study followed standard GT-seq methods that implemented two rounds of polymerase chain reaction (PCR) to first amplify targeted SNPs and then add dual barcodes for the identification of all individuals in downstream analyses. Following the barcoding step, the concentration of each sample was normalized and then pooled into a library. Once the library was prepared, it was sequenced on an Illumina NextSeq 500 instrument and then was genotyped with scripts from Campbell et al. (2015). As a quality control step, all samples and loci with ≥10% missing genotypes were removed from further analyses. Genotype data were retained when >90% loci successfully genotyped and had an estimated <0.5% genotyping error based on replicate genotyping of 10% of samples.

To select the final samples for the whole-genome resequencing step, samples were first analyzed and filtered according to biases from kinship, population structure, and sex imbalances among samples. The sex of individuals was determined with the *O. mykiss* sex marker (OmyY1_2SEXY; Brunelli et al., 2008) with the intent to split individuals into four groups (resident male, anadromous male, resident female, and anadromous female) for statistical analysis. This approach was intended to account for sex specific differences in migration behavior and isolate confounding signals of sex-linked genes including the known sdY region on chromosome Omy29 (Gao et al., 2021). Kinship and population biases were tested at neutral loci with a compressed linear model (Zhang et al., 2010) that used a kinship matrix produced with the VanRaden algorithm (VanRaden et al., 2008) along with three principal components and were implemented in Genome Association and Prediction Integrated Tool (GAPIT-R package; Lipka et al., 2012).

Our approach to genome resequencing was to target low read depth for individually barcoded samples and then to combine sequence reads from individuals of the same phenotype for further analyses of the four groups: resident male, anadromous male, resident female, and anadromous female. To prepare the libraries for whole-genome resequencing, individuals were barcoded, and a NEBNext Ultra enzymatic fragmentation protocol was followed (Horn et al., 2020). Briefly, we extracted DNA according to a Chelex extraction method (Sweet et al., 1996). Individual sample DNA concentrations were quantified with pico-green fluorescence on a Tecan M200 (Männedorf, Switzerland) to normalize the quantity of DNA from each sample within two standard deviations of the mean concentration. After barcoding individuals, the DNA samples were combined to avoid batch effects and normalized DNA was fragmented with NEBNext double-stranded DNA fragmentase, which fragments randomly throughout the genome. Fragments of 400–600 base pairs (bp) were size-selected, and then, ends of DNA strands were repaired with NEBNext end prep. NEBNext adaptors were ligated to the repaired and size-selected fragments and cleaned with SpriSelect Beads. We implemented PCR amplification and cleaned the PCR product with SpriSelect Beads. The quantity and quality of each library were assessed using quantitative PCR and a Tape Station with a DNA High Sensitivity D1000 kit and then normalized prior to sequencing. Final libraries were sequenced on an Illumina NextSeq 500 sequencer, targeting 500 million paired-end reads per library.

### 2.3 Statistical Analyses

Barcodes were useful to normalize sequence reads for individual samples, but samples were subsequently pooled for analyses by migratory traits to provide adequate sequencing depth to identify SNPs and estimate allele frequencies for each phenotypic group. The whole-genome resequencing data were analyzed with a pipeline, called PoolParty (Micheletti and Narum 2018), that can handle either pooled or individually barcoded sequence data. The PoolParty pipeline consists of bash scripts that utilize open-source packages to align to a reference genome and filter raw whole-genome resequencing data. In this study, sequence reads were aligned to *O. mykiss* reference assembly GCA_013265735.3 (Gao et al., 2021). Next, the pipeline assesses filtered alignments with BBMap (Bushnell 2016), bwa mem (Li 2013), samblaster (Faust and Hall 2014), Picard Tools (Broad Institute), and bcftools (Li et al., 2011). Mapped-read coverage statistics were determined to evaluate mapping efficiency to the reference genome with mean depth of coverage and proportion of the reference assembly genome covered by mapped reads and filtered accordingly. To filter the SNPs that were used in downstream analyses, we assessed coverage and minor allele frequency (MAF). When SNPs had coverage either below 15X or above 100X, or MAF below 0.05, they were filtered out of analyses. Pairwise analyses, such as the fixation index (F_ST_), sliding window FST (sFST), Fisher’s exact test (FET), and local score (Fariello et al., 2017), were conducted for resident versus anadromous groups with Popoolation2 scripts (Kofler et al., 2011). To determine significance of difference between SNPs in resident and anadromous groups, we used false discovery rate (FDR) corrected *p*-values from FET analyses of allele frequencies for individual SNPs. A QQ plot analysis was conducted to evaluate the potential bias of association tests and effects of covariates.

To test for polygenic signals of association with anadromy, we analyzed the data set with an R package called Random Forest (RF; Liaw and Wiener 2002). This RF package is a machine learning algorithm that provides a nonparametric framework to identify epistasis through identification of a group of loci predictive of phenotypic traits with significant differences in allele frequencies that better predict the trait than a single locus (Brieuc et al., 2018). To detect these loci, a “forest” of classification or regression trees is partitioned recursively by randomly sampling, with replacement, creating a training subset of data, and a random selection of a subset of predictors to find which partition of loci best predicts the trait by minimizing within-group variance or error. The RF algorithm first identifies the best predictive loci partition as the first node of the “tree” and then continues to randomly subset the remaining predictors. Selections of partitions were random to avoid bias and curtail differences within and between the “trees” (Goldstein et al., 2011; Rokach 2016). An error rate, a proportion of the variation explained (PVE), and an importance value were calculated with a subset of samples left out of the “tree” building steps as training data. The downside to random selections is that, if an important predictor is removed, then the predictive power of the “tree” will decrease; this can be belied with analyses of many “trees” and importance values of partitions across the “forest,” instead of relying too heavily upon a single “tree”.

To examine previously identified candidate genes for anadromy, we analyzed genotypes from candidate markers that were included in the GT-seq panel across phenotypic groups. This included several markers from the genome region described by Pearse et al. (2019) for large inversions on chromosome Omy05 of coastal California *O. mykiss* populations associated with the anadromy phenotype. In addition, we tested the three candidate loci from Narum et al. (2011) that had previously shown significant association with anadromy in previous analyses in *O. mykiss* from the Klickitat River. For these candidate markers that were genotyped with GT-seq, we assessed the MAF across all individuals and tested for differences in allele and genotype frequencies for anadromous and resident groups with FDR correction for multiple tests.

## 3 Results

**FIGURE 2:**
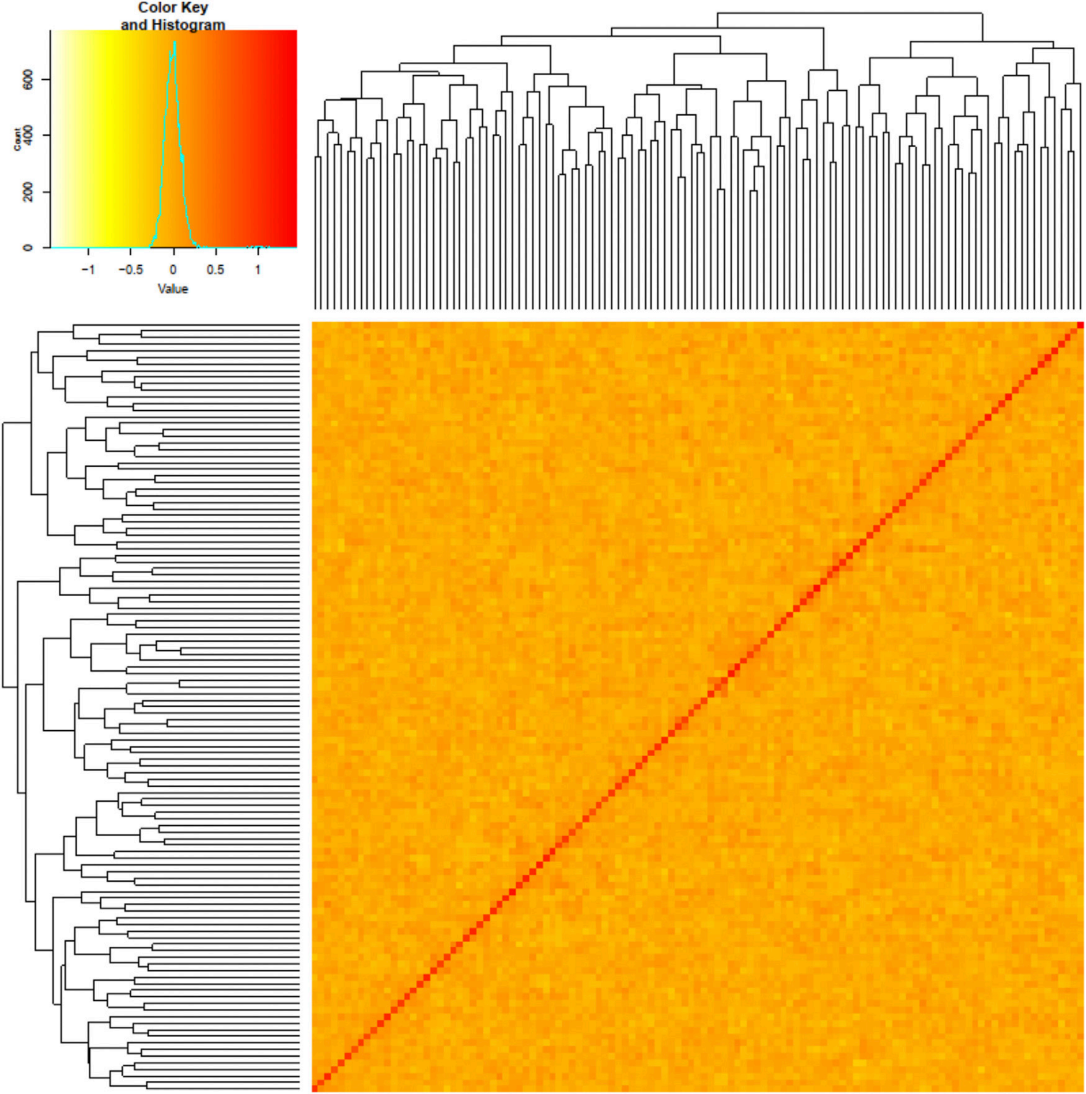
Kinship matrix for 117 samples with darker colors and higher kinship values (e.g., kinship values ≥ 1 in this population) representing higher kinship between individuals. The diagonal represents relatedness values of each individual to itself as a reference for high kinship patterns. A phylogram of individual relatedness among samples is shown on both axes.

Results from genotyping the initial GT-seq panel were used to select the individuals for whole-genome resequencing. For the samples genotyped with GT-seq, the average genotype error rate was 0.51% and individuals missing ≥10% genotypes were excluded from analyses. Of the 198 individuals genotyped, three failed, leaving 195 for further analyses. Of those 195 individuals, the marker for genetic sex identified 117 males (78 resident and 39 anadromous) and 78 females (3 resident and 75 anadromous). This result reflected lower variation in migration life history for females than males (Supplementary Table S1) and a limited sample size of resident females that excluded intended analyses within the female groups (resident vs. anadromous). Analyses for potential kinship bias or population structure revealed minimal signal among the samples (Figure 2), and no individuals were excluded from further analyses.

**FIGURE 3:**
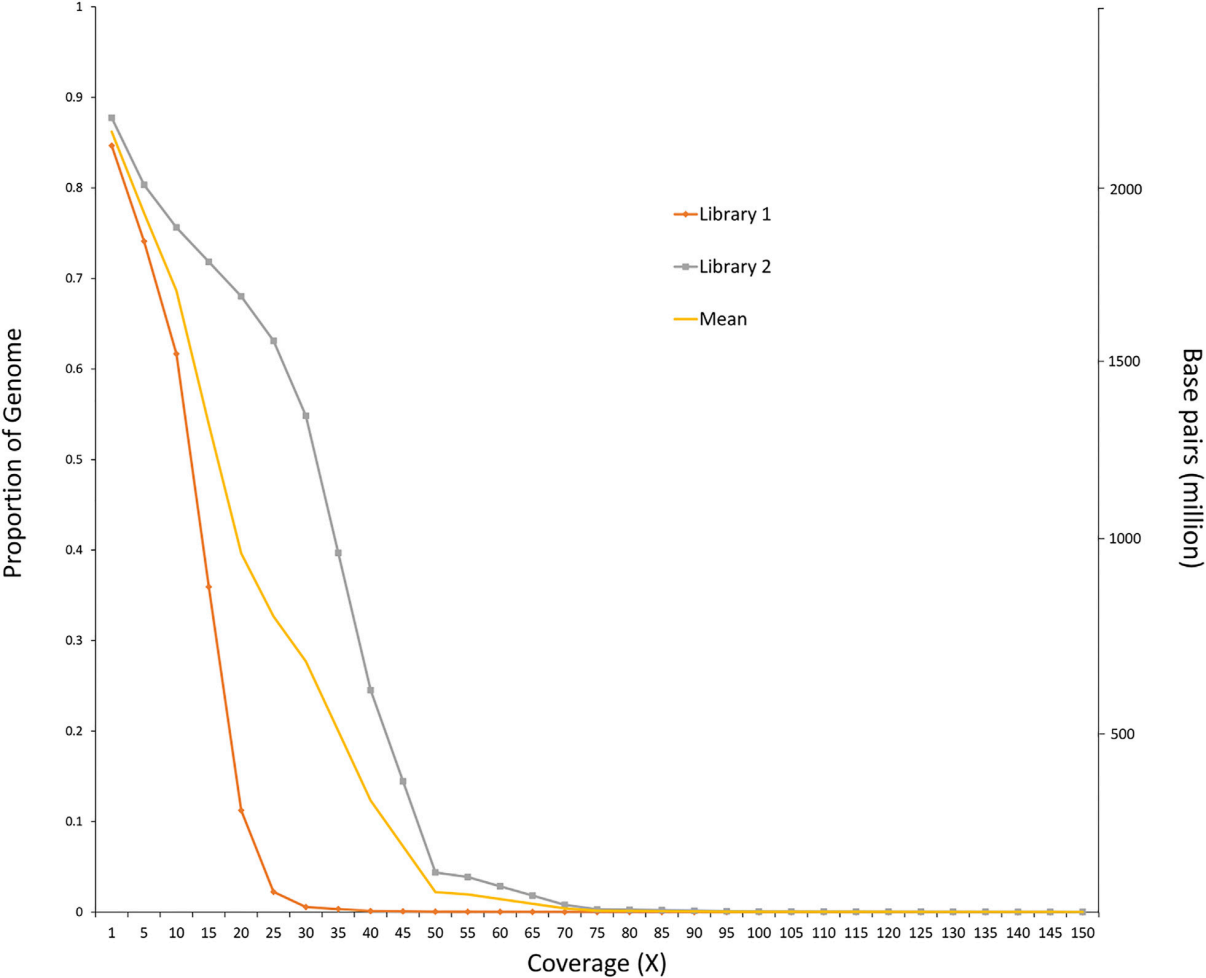
Proportion of genome covered at different sequence read depths. The orange line represents Library 1, which consists of 39 anadromous males. The gray line represents library 2, which consists of 78 resident males.

**FIGURE 4:**
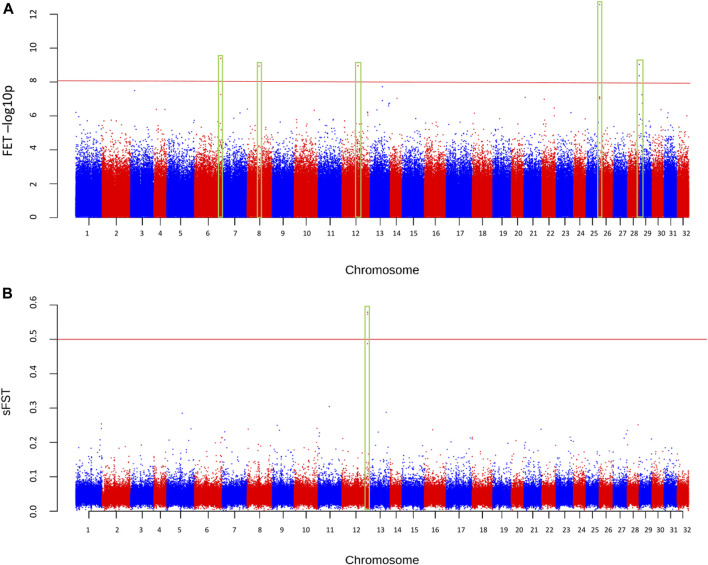
Manhattan plot of differences in allele frequencies between resident and anadromous collections across the genome based on **(A)** significance from Fisher’s exact test (FET), and **(B)** sliding window F_ST_ (sF_ST_). The minimum coverage threshold was 15 reads and the minor allele frequency was > = 0.05.

The final sample set included 117 male *O. mykiss* that were split into two phenotypic groups with 39 anadromous (4 returning to spawn and 35 out-migrating) steelhead and 78 resident rainbow trout. Because there were insufficient resident females (*n* = 3) to test for migratory differences within females (resident vs. anadromous), an attempt was made to combine females with males in their respective phenotypic group even though this caused a severe imbalance in sex ratio between groups of residents (3 female, 78 male; 3.7% female) versus anadromous (75 female, 39 male; 67.8% female). Imbalanced sex ratios in analyses were expected to lead to detection of sex-linked regions as demonstrated in previous studies (Benestan et al., 2017) but were completed for thoroughness. The average sequence coverage for the anadromous males was 0.36× per individual and was 0.43× per individual for the resident males (Figure 3). After filtering, a total of 5.64 million SNPs were included for further tests for differences in allele frequency between phenotypic groups. Analyses with the PoolParty pipeline included results for F_ST_, FET, sliding window F_ST_, and local score analyses. Analyses of allele frequencies among the resident and anadromous groups indicated that six markers on multiple chromosomes were significant after Bonferroni correction with FET (Figure 4A) including those on chromosomes Omy06, Omy08, Omy12, Omy26, and Omy29 (Table 1). However, only SNPs on chromosome Omy12 were significant among groups with sliding window F_ST_ and were considered the strongest candidate region for anadromy (Figure 4B). Significant SNPs on chromosome Omy12 were located 28.6–43.6 kb from the nearest gene, NACHT, LRR, and PYD domain–containing protein 3 (NLRP3), detected with the sliding window F_ST_ analysis (Table 1). The gene positioned closest (10.9 kb) to the significant SNP detected with FET analysis on chromosome Omy12 was the COP9 signalosome complex subunit 6 (CSN6) (Table 1). Analyses of combined sexes for resident vs. anadromous (with highly imbalanced sex ratios) yielded a strong signal near the sdY sex determining region on chromosome Omy29 as expected (Supplementary Figure S5).

**TABLE 1:** Genomic position and closest gene annotations for significant markers from Fisher's Exact Test (FET) and sliding window Fst (sFst) analyses on chromosome (Chr) Omy12.

SNP	Chr	Position	FET *p*-value value	sF_ST_ Differentiation value	Source	Closest Gene(s)	Possible Gene Function
1326266	6	95913987	4.03E-10	NA	PoolParty - fet	Leucine-rich repeat extensin-like protein 1	Leucine-rich repeat
1661566	8	43382768	1.14E-09	NA	PoolParty - fet	MYC proto-oncogene, bHLH transcription factor b	Leucine zipper, Myc, Myc-type, basic helix-loop-helix (bHLH) domain, transcription regulator Myc, N-terminal
2557802	12	58504189	1.10E-09	NA	PoolParty - fet	COP9 signalosome complex subunit 6	Posttranslational modification, protein turnover, chaperones, cell wall/membrane/envelope biogenesis
4778210	26	1643096	2.62E-13	NA	PoolParty - fet	Calcineurin-like phosphoesterase domain, ApaH type	Carbohydrate transport and metabolism, posttranslational modification, protein turnover, chaperones, mobilome, prophages, transposons
5119622	29	77226	8.99E-10	NA	PoolParty - fet	Anoctamin	Protein dimerization activity, integral component of membrane
5119623	29	77228	4.23E-09	NA	PoolParty - fet	Anoctamin	Protein dimerization activity, integral component of membrane
204161	12	93625000	NA	0.487	PoolParty - Sfst	NACHT, LRR, and PYD domain–containing protein 3	Leucine-rich repeat, transcription, signal transduction mechanisms, inorganic ion transport and metabolism
204162	12	93630000	NA	0.573	PoolParty - Sfst	NACHT, LRR, and PYD domain–containing protein 3	Leucine-rich repeat, transcription, signal transduction mechanisms, inorganic ion transport and metabolism
204163	12	93635000	NA	0.579	PoolParty - Sfst	NACHT, LRR, and PYD domain–containing protein 3	Leucine-rich repeat, transcription, signal transduction mechanisms, inorganic ion transport and metabolism
204164	12	93640000	NA	0.579	PoolParty - Sfst	NACHT, LRR, and PYD domain–containing protein 3	Leucine-rich repeat, transcription, signal transduction mechanisms, inorganic ion transport and metabolism

Further analyses that account for physically linked SNPs (local score) or polygenic association (RF) provided no consistent signals of association and thus were considered false positive or unreliable results. Analyses with local score revealed many small but significant peaks on every chromosome of the genome (Supplementary Figure S1). Because local score is highly susceptible to false positives due to multiple testing effects of linked SNPs, a stringent critical value (*p*-value < 0.001 after Bonferroni correction) was applied and indicated that significant peaks occurred on several chromosomes (Supplementary Figure S2). We also searched for polygenetic association for epistatic loci with RF methods and significant loci were detected in multiple trees. However, candidates were not repeatable across replicate trees in the analyses and thus were discounted as reliable signals of polygenic association (Supplementary Figure S4). The QQ plot displayed most SNPs falling along the line representative of the null hypothesis or SNPs with no association to the anadromy trait (Supplementary Figure S6). The QQ plot also had loci that display deviation from the null hypothesis line, including every chromosome Omy12 marker identified with the other analyses, which were clustered even further from the null hypothesis line and above the stringent significance threshold of observed sF_ST_ > 0.5 (Supplementary Figure S6).

Results for the 12 markers in the inversion complex of chromosome Omy05 (Pearse et al., 2019) revealed that several were nearly fixed with very low variation in our samples, and others had no significant differences in allele frequencies (Table 2). The Omy_u09-61.043 marker was fixed for the resident or derived allele, but this was not the case for all markers in this inversion (Table 2). Of the three candidate markers from Narum et al. (2011), two had similar allele frequencies between resident and anadromous groups, and none were significant after a FDR correction (Benjamini and Hochberg 1995). However, the marker with the lowest *p*-value (*p* = 0.134; Omy_ndk-152; Table 2) had an allele frequency difference of 9% between resident and anadromous groups and is located at position 59,538,315 on chromosome Omy12. This is near the region on chromosome Omy12 that was found to be significant in the whole-genome analyses.

**TABLE 2:** Chromosome (Chr) Omy05 markers from Pearse et al.(2019) and candidate genetic markers from Narum et al. (2011) were analyzed with minor allele frequencies (MAF) and Fisher's Exact Test (FET) for the samples for this study.

Genetic marker name	Chr	Total MAF	Anadromous MAF	Resident MAF	Homozygous MAF	Heterozygous MAF	Homozygous Major Allele Frequency	Inversion	FET *p*-value
OmyR19198Pearse	5	0.021	0.026	0.019	0	0.043	0.957	1	1
OmyR40252Pearse	5	0.021	0.026	0.019	0	0.043	0.957	1	1
OmyR14589Pearse	5	0.021	0.026	0.019	0	0.043	0.957	1	1
Omy_RAD23894-58	5	0.137	0.179	0.115	0.009	0.256	0.735	1	0.202
OmyR24370Pearse	5	0.021	0.026	0.019	0	0.043	0.957	1	1
Omy_bcAKala-380th	5	0.462	0.449	0.468	0.248	0.427	0.325	2	0.799
OmyR33562Pearse	5	0.021	0.026	0.019	0	0.043	0.957	2	1
Omy_u09-61.043	5	0.000	0.000	0.000	0.000	0.000	1.000	2	No score, fixed
Omy_RAD30392-17	5	0.402	0.385	0.410	0.214	0.376	0.393	2	0.699
Omy_SECC22b-88	5	0.009	0.000	0.013	0	0.017	0.983	2	0.551
OmyR40319Pearse	5	0.004	0.000	0.006	0	0.009	0.991	2	1
Omy_ndk152	12	0.177	0.244	0.154	0.017	0.333	0.632	NA	0.13444
Omy_IL6320	14	0.177	0.205	0.173	0.060	0.248	0.692	NA	0.62277
Omy_LDHB2_i6	21	0.115	0.115	0.115	0.017	0.205	0.761	NA	0.83429

## 4 Discussion

In this study, we conducted multiple analyses to test for genomic regions of high differentiation between the resident and anadromous samples and examine consistent signals that would identify candidate genes associated with this life history trait. Tracking migratory behavior of PIT-tagged juveniles provided resident vs. anadromous (outmigration) phenotypes for genomic analyses, and distinct patterns in migration behavior were observed by each sex. The natural origin *O. mykiss* captured in the Klickitat River screw trap in 2018, 2019, and 2021 were classified as smolts due to distinct physical characteristics at rates of 99.5% (*n* = 3,615), 99.4% (*n* = 3,475), and 99.7% (*n* = 2,352), respectively. Outmigrating individuals detected in the Columbia River after leaving the Klickitat River were most likely steelhead smolts, but downstream migrating resident rainbow trout cannot be completely ruled out. Females rarely remained as residents in contrast to males despite collection from the same stream reaches, which is consistent with the differences in migratory behavior among sexes that has been generally observed in previous studies of this species (Fleming and Reynolds 2004; Quinn et al., 2011). The lack of female resident fish limited genomic analyses to largely focus on variation in male migratory phenotypes, but results revealed a candidate region of minor effect on chromosome Omy12.

Analyses with several approaches were considered necessary to account for limitations of each method and increase confidence in potential candidate genes. For example, F_ST_ and sliding window F_ST_ have been demonstrated to be susceptible to false positives or skewed signal to noise (Fariello et al., 2017), and thus, local score was applied from FET results to improve signal to noise in this high-density genome scan. The RF method was expected to be more effective to detect polygenetic signals of minor effect genes across the genome, especially for natural populations with low linkage disequilibrium (LD) (Holliday et al., 2012). However, analyses from this study yielded highest confidence from tests of sliding window F_ST_ and significance based on FET, whereas other results appeared to suffer from high levels of false positives.

Analyses provided highest confidence for a genomic region of minor effect on chromosome Omy12 for anadromy in male steelhead in the Klickitat River. We chose to align resequencing data from this study to the highest quality genome assembly that was currently available for the species (Gao et al., 2021). Although karyotypes vary across lineages of *O. mykiss*, the number of chromosome arms is constant (Thorgaard et al., 1983; Phillips et al., 2006; Pearse et al., 2019). The newest Arlee assembly has 32 haploid chromosomes due to fissions at chromosomes Omy04, Omy14, and Omy25, but chromosome Omy12 demonstrates homology between Arlee and Swanson lines (Gao et al., 2021). Thus, we do not expect that results observed at Omy12 in the current study were due to problems in homology with the reference genome assembly. The gene nearest to significant SNPs was CSN6 has been shown to be overexpressed in a variety of cancer specimens (Zhang et al., 2013). Another nearby gene on chromosome Omy12 was the NLRP3, which has previously been observed functioning to restrict bacterial infection in Japanese flounder (Chen et al., 2020). The function of these genes did not bear an obvious biological connection to resident or anadromous behaviors and would require further validation before speculating on their related functions in *O. mykiss*.

Six markers specifically developed from the large inversion region on chromosome Omy05 (Pearse et al., 2019) had nearly zero variation among samples in this study and thus were not informative for analyses. This is consistent with results from Pearse et al. (2019) that observed significant results at chromosome Omy05 for coastal populations from the southern portion of the range but were not informative in other portions of the species’ range. Additional markers that map to the same region on chromosome Omy05 that were developed in previous restriction site-associated DNA studies (Micheletti and Narum 2018) were variable but not significantly different between the two life history types in the Klickitat River. The difference in variation from markers in the chromosome Omy05 region was unexpected because there is extensive LD across the entire double inversion (Pearse et al., 2019), but this may reflect near fixation of derived alleles followed by subsequent recombination in this population that is part of the distinct inland lineage of *O. mykiss* (e.g., Collins et al., 2020). Previous candidate markers for anadromy in *O. mykiss* from the Klickitat River (Narum et al., 2011) were also not significant, but it was notable that one marker (Omy_ndk-154) was relatively near the same region on chromosome Omy12 identified in the whole-genome scan and had modest difference in allele frequencies between life history types. However, this marker may be too distant (∼37 Mb) from the candidate region on chromosome Omy12 to be informative to distinguish life history variation. Thus, there was no evidence in this study population for association with Omy05 migratory behavior as has been shown in a previous study (Pearse et al., 2019). When the results of this study were compared to similar studies (Narum et al., 2011; Hecht et al., 2012; Hale et al., 2013; Hecht et al., 2013; Campbell et al., 2021), a significant association with anadromy was found on chromosome Omy12 in all cases, but these studies have consistently suggested polygenic association located throughout the genome.

Overall, analyses conducted on the *O. mykiss* samples collected from the Klickitat River for this study detected loci significantly associated with the trait of anadromy that may represent candidate genes of minor effect within this *O. mykiss* population. The significant region on chromosome Omy12 provided the strongest support; however, this region contains multiple candidate genes with uncertain biological relevance to resident versus anadromous life history types. Thus, candidate genes of major effect for anadromy remain elusive and results continue to support the hypothesis that the genetic basis for life history types in this lineage of *O. mykiss* is highly polygenic with minor effect (e.g., Nichols et al., 2008; Narum et al., 2011; Hecht et al., 2012; Hale et al., 2013; Hecht et al., 2013; Campbell et al., 2021). Previous studies evaluated gene expression associated with anadromy at various stages of development of anadromous steelhead and resident rainbow trout and uncovered differentially expressed genes throughout the genome, including many on chromosome Omy12 (McKinney et al., 2015; Hale et al., 2016). In addition, Baerwald et al. (2016) evaluated differentially methylated regions (DMRs) to assess the role of epigenetics on smoltification in *O. mykiss* and identified one DMR associated with smoltification on chromosome Omy12. It is also likely that the main drivers of anadromy in this lineage of *O. mykiss* may be more strongly related to environmental rearing such as epigenetic modification, gene expression, and phenotypic plasticity. Future studies into these various mechanisms regulating anadromy are needed to further understand variation in the life history of this protected species.

## Data Availability

The original contributions presented in the study are publicly available. This data can be found here: PRJNA771561.
