# Supplementary figures and images for: Whole-Genome Resequencing to Evaluate Life History Variation in Anadromous Migration of Oncorhynchus mykiss
Source: Front Genet. 2022 Mar 15;13:795850. doi: 10.3389/fgene.2022.795850 (PMC8964970; doi:10.3389/fgene.2022.795850)

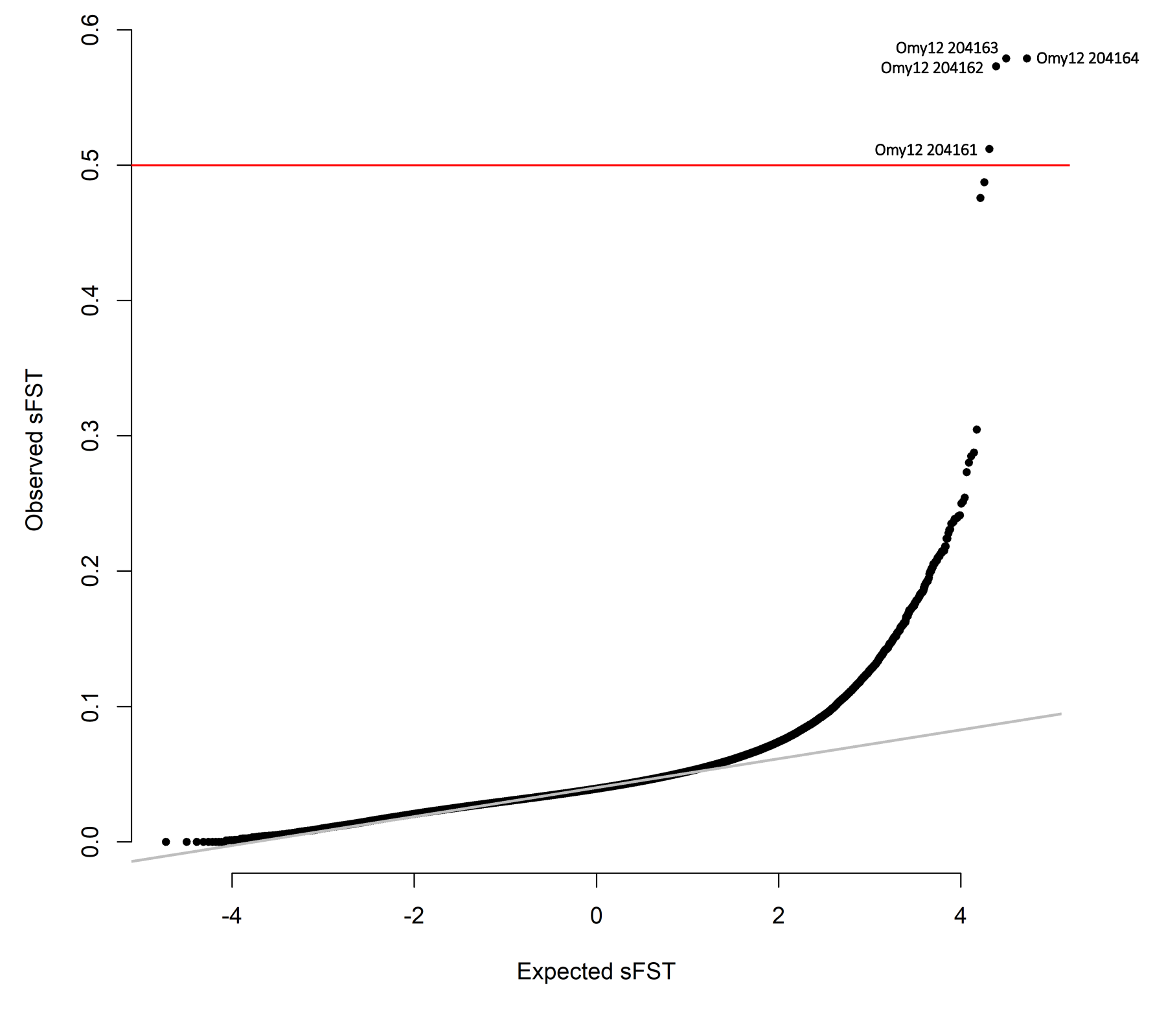

Supplement: Supplementary file 1 [file Image6.TIF]

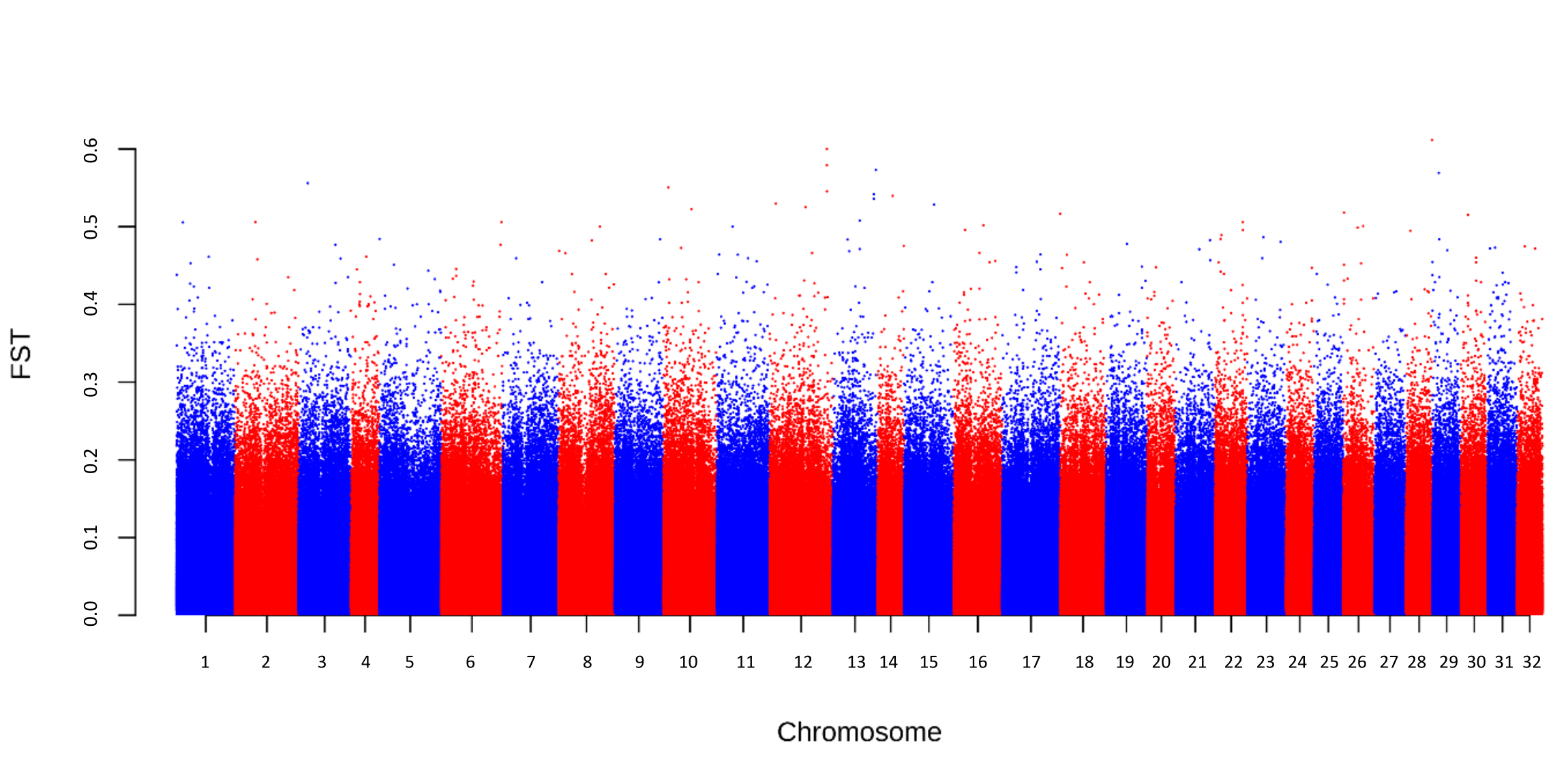

Supplement: Supplementary file 3 [file Image3.TIF]

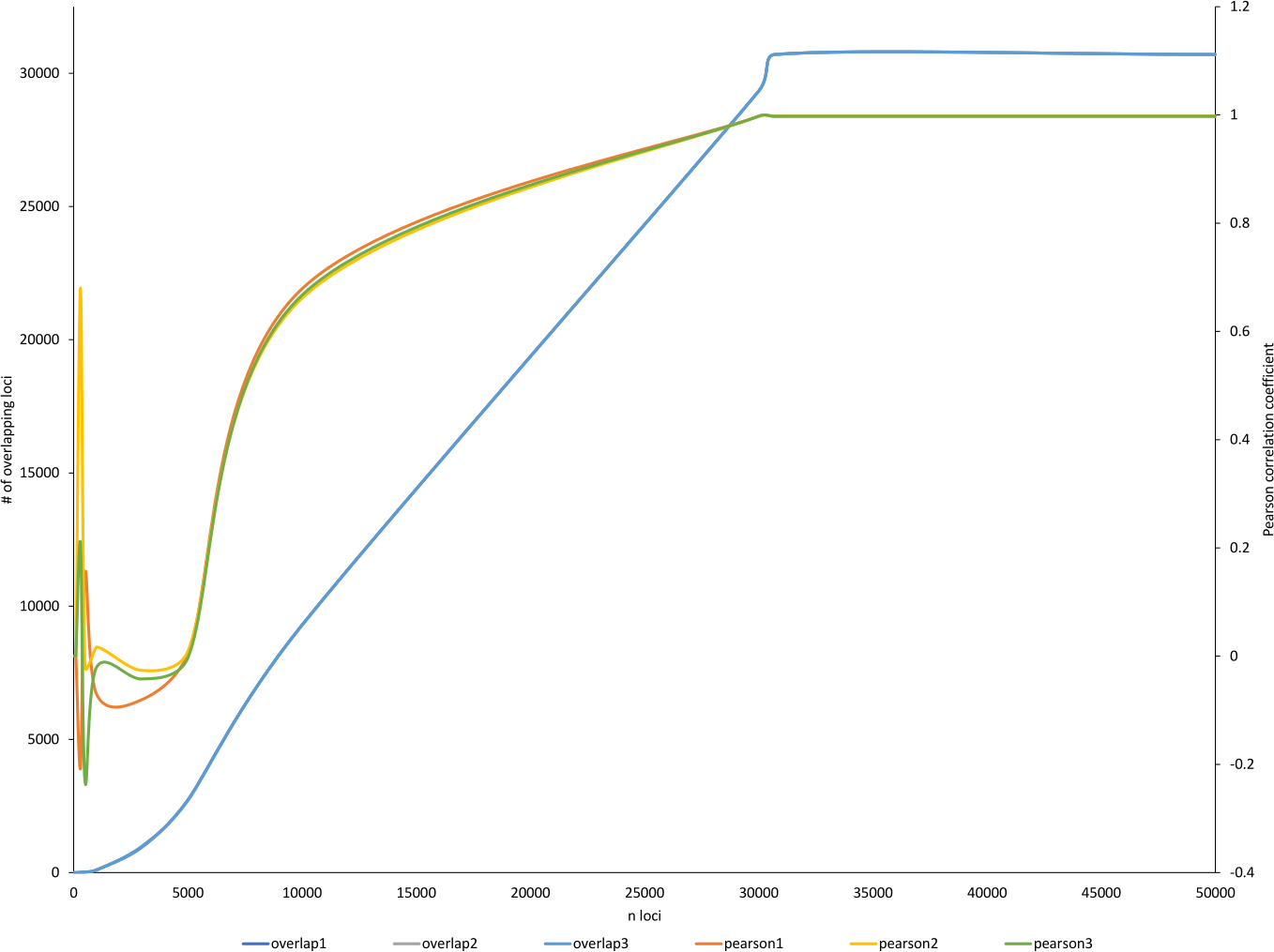

Supplement: Supplementary file 4 [file Image4.TIF]

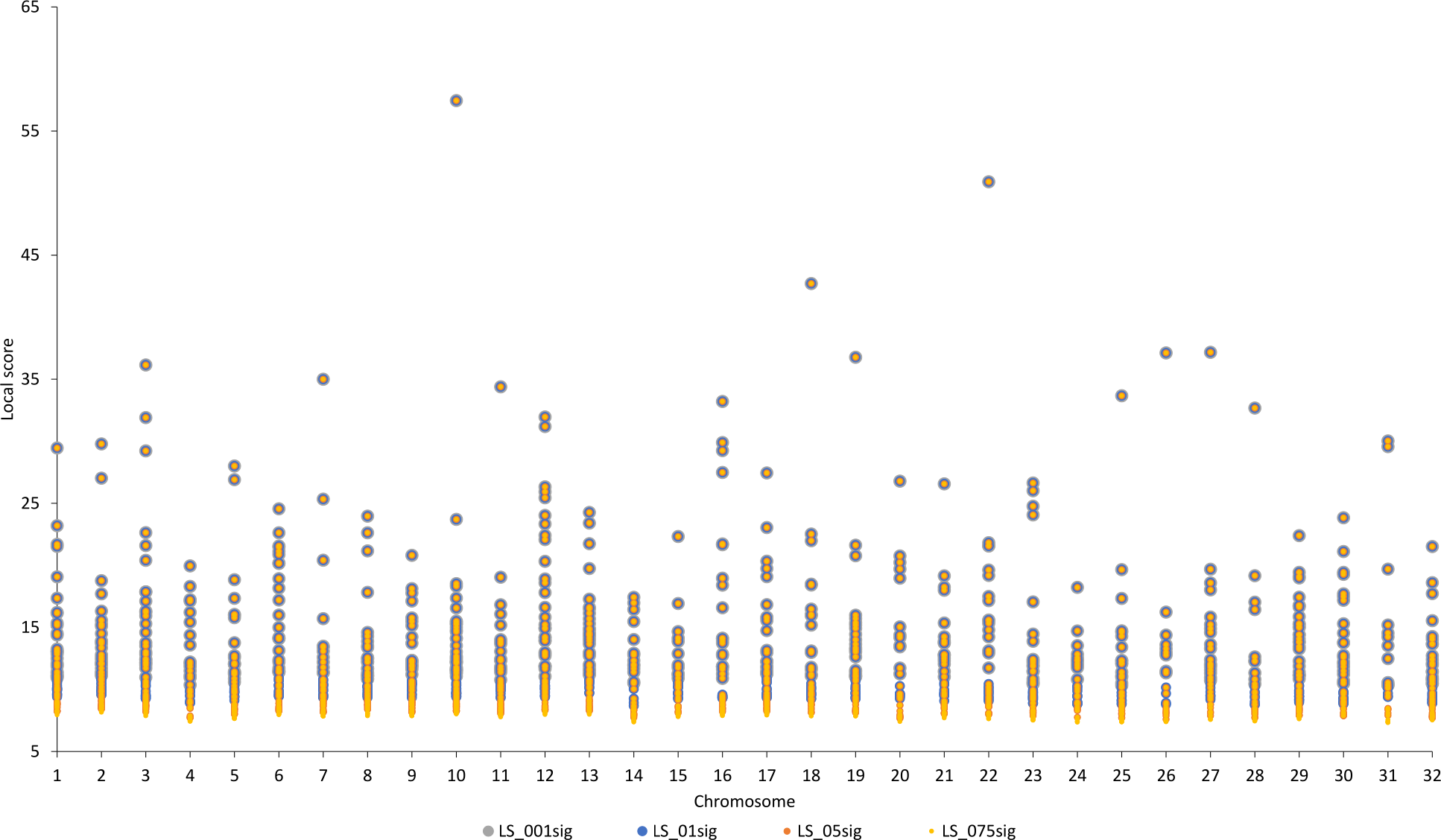

Supplement: Supplementary file 5 [file Image2.TIF]

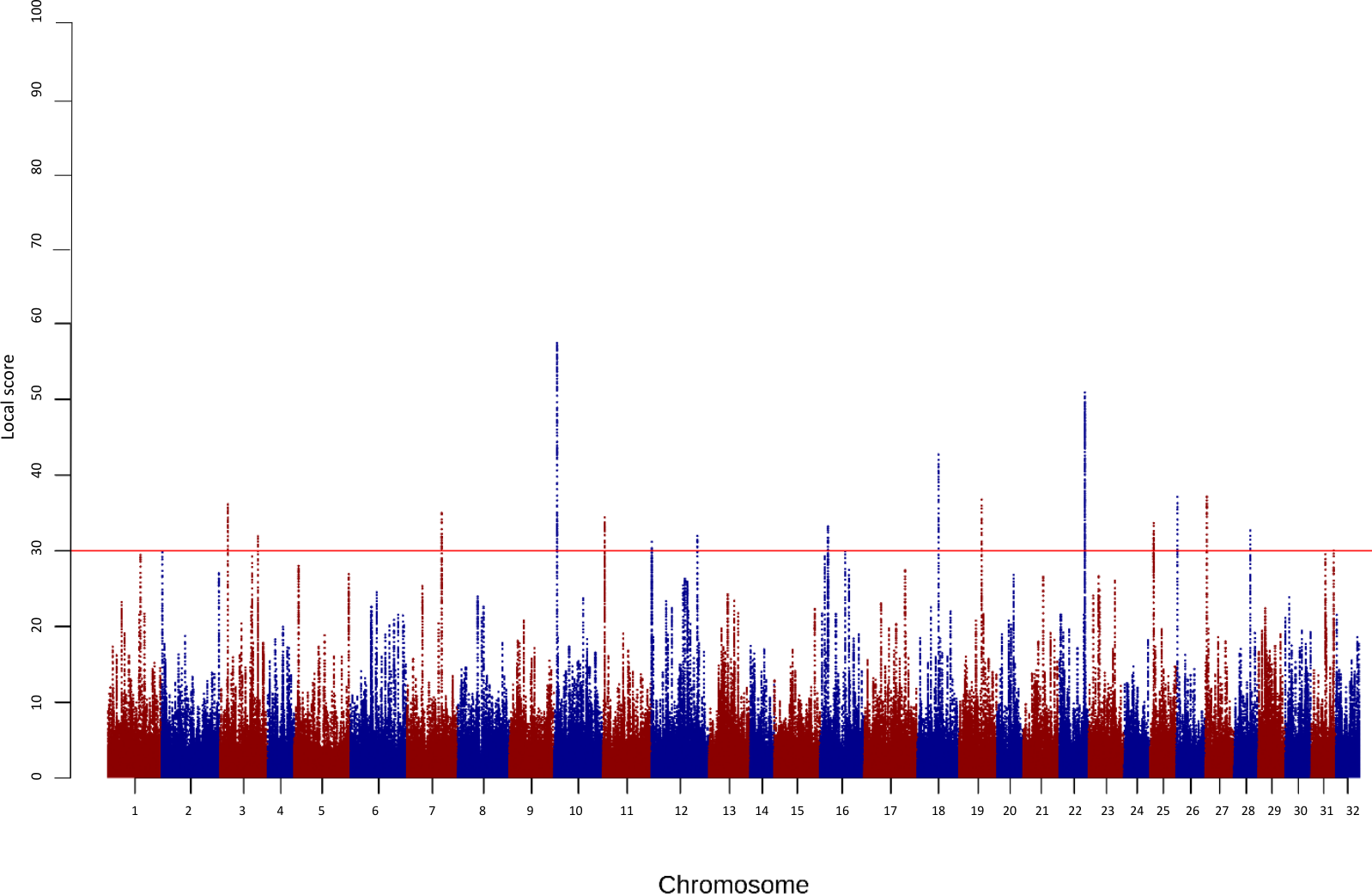

Supplement: Supplementary file 6 [file Image1.TIF]

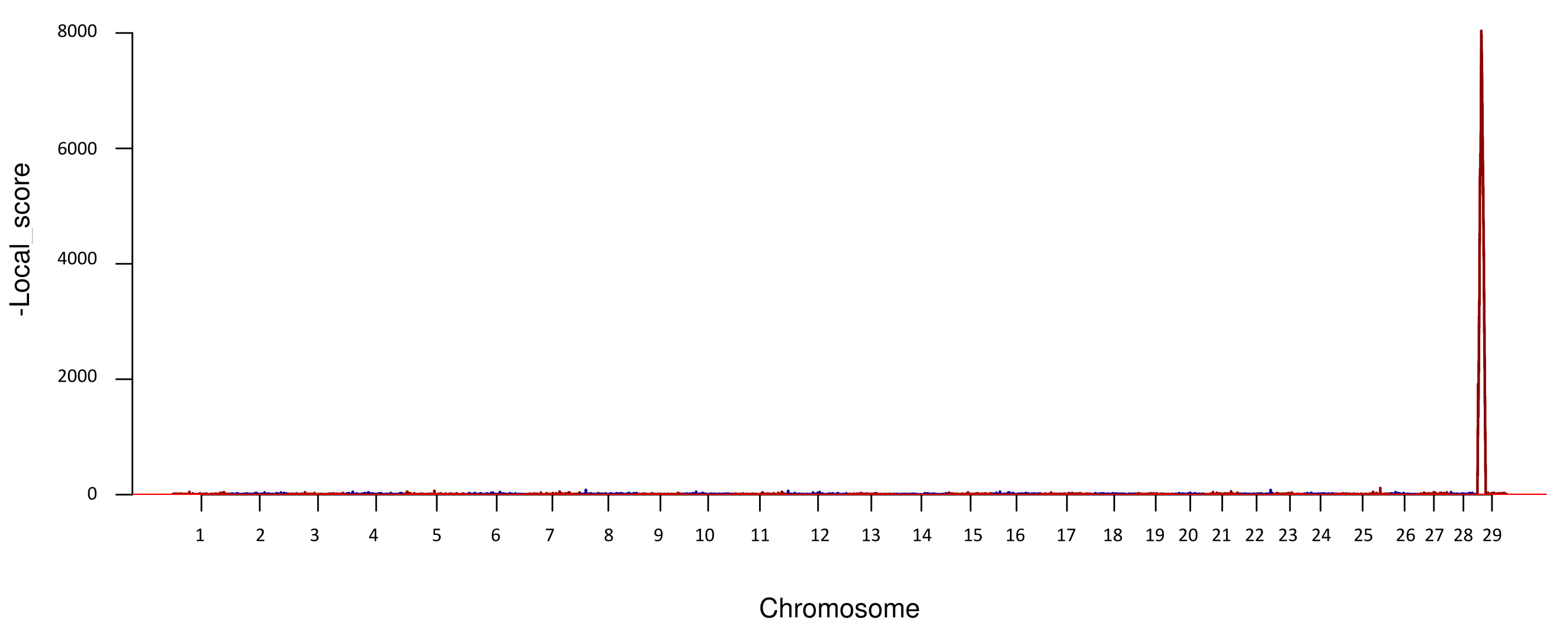

Supplement: Supplementary file 8 [file Image5.TIF]
