# Supplementary material for: Whole-Genome Resequencing to Evaluate Life History Variation in Anadromous Migration of Oncorhynchus mykiss
Source: Front Genet. 2022 Mar 15;13:795850. doi: 10.3389/fgene.2022.795850 (PMC8964970; doi:10.3389/fgene.2022.795850)
Supplement: Supplementary file 2 [file Table1.DOCX]

**Supplementary Table 1.** Sample names, life history types, sample tributary, and years.

| **Sample Name** | **Life history type** | **Tributary** | **Latitude** | **Longitude** | **Year** | **Sex** | **Retained for final analyses?** |
| --- | --- | --- | --- | --- | --- | --- | --- |
| OmyKR11-BRC08-C1 | Anadromous | Brush Creek | 46.163 | -120.945 | 2011 | M | Y |
| OmyKR11-BRC08-C2 | Anadromous | Brush Creek | 46.163 | -120.945 | 2011 | F | N |
| OmyKR11-BRC08-C3 | Resident | Brush Creek | 46.163 | -120.945 | 2011 | M | Y |
| OmyKR11-BRC08-E8 | Resident | Brush Creek | 46.163 | -120.945 | 2011 | M | Y |
| OmyKR11-BRC08-F2 | Anadromous | Brush Creek | 46.163 | -120.945 | 2011 | F | N |
| OmyKR11-BRC08-G2 | Resident | Brush Creek | 46.163 | -120.945 | 2011 | M | Y |
| OmyKR11-BRC08-I5 | Resident | Brush Creek | 46.163 | -120.945 | 2011 | M | Y |
| OmyKR11-BRC38-A3 | Resident | Brush Creek | 46.163 | -120.945 | 2011 | M | Y |
| OmyKR11-BRC41-H5 | Resident | Brush Creek | 46.163 | -120.945 | 2011 | M | Y |
| OmyKR11-ETC13-B3 | Resident | EF Tepee Creek | 46.155 | -121.020 | 2011 | M | Y |
| OmyKR11-TPC10-B5 | Anadromous | Tepee Creek | 46.181 | -121.025 | 2011 | F | N |
| OmyKR11-TPC10-C9 | Anadromous | Tepee Creek | 46.181 | -121.025 | 2011 | M | Y |
| OmyKR11-TPC10-E1 | Anadromous | Tepee Creek | 46.181 | -121.025 | 2011 | M | Y |
| OmyKR11-TPC10-E3 | Anadromous | Tepee Creek | 46.181 | -121.025 | 2011 | M | Y |
| OmyKR11-TPC11-C1 | Resident | Tepee Creek | 46.181 | -121.025 | 2011 | M | Y |
| OmyKR11-TPC11-G9 | Resident | Tepee Creek | 46.181 | -121.025 | 2011 | M | Y |
| OmyKR11-TPC12-B6 | Resident | Tepee Creek | 46.181 | -121.025 | 2011 | M | Y |
| OmyKR11-TPC17-A1 | Resident | Tepee Creek | 46.181 | -121.025 | 2011 | M | Y |
| OmyKR11-TPC17-C6 | Resident | Tepee Creek | 46.181 | -121.025 | 2011 | M | Y |
| OmyKR11-TPC17-E4 | Resident | Tepee Creek | 46.181 | -121.025 | 2011 | M | Y |
| OmyKR11-TPC20-C2 | Resident | Tepee Creek | 46.181 | -121.025 | 2011 | M | Y |
| OmyKR11-TPC21-A10 | Anadromous | Tepee Creek | 46.181 | -121.025 | 2011 | F | N |
| OmyKR11-TPC21-A4 | Anadromous | Tepee Creek | 46.181 | -121.025 | 2011 | F | N |
| OmyKR11-TPC22-C4 | Anadromous | Tepee Creek | 46.181 | -121.025 | 2011 | F | N |
| OmyKR11-TPC23-A2 | Resident | Tepee Creek | 46.181 | -121.025 | 2011 | M | Y |
| OmyKR11-TPC24-A6 | Resident | Tepee Creek | 46.181 | -121.025 | 2011 | M | Y |
| OmyKR11-TPC24-B1 | Resident | Tepee Creek | 46.181 | -121.025 | 2011 | M | Y |
| OmyKR11-TPC24-B4 | Resident | Tepee Creek | 46.181 | -121.025 | 2011 | M | Y |
| OmyKR11-TPC24-B5 | Resident | Tepee Creek | 46.181 | -121.025 | 2011 | M | Y |
| OmyKR11-TPC24-D3 | Resident | Tepee Creek | 46.181 | -121.025 | 2011 | M | Y |
| OmyKR11-TPC37-A8 | Anadromous | Tepee Creek | 46.181 | -121.025 | 2011 | F | N |
| OmyKR11-TPC37-B6 | Resident | Tepee Creek | 46.181 | -121.025 | 2011 | M | Y |
| OmyKR11-TPC48-B7 | Resident | Tepee Creek | 46.181 | -121.025 | 2011 | M | Y |
| OmyKR11-TPC50-B10 | Anadromous | Tepee Creek | 46.181 | -121.025 | 2011 | M | Y |
| OmyKR11-WHC09-A10 | Resident | White Creek | 46.013 | -121.150 | 2011 | M | Y |
| OmyKR11-WHC15-G5 | Anadromous | White Creek | 46.013 | -121.150 | 2011 | F | N |
| OmyKR11-WHC18-A5 | Resident | White Creek | 46.013 | -121.150 | 2011 | M | Y |
| OmyKR11-WHC19-B1 | Anadromous | White Creek | 46.013 | -121.150 | 2011 | F | N |
| OmyKR11-WHC19-D3 | Anadromous | White Creek | 46.013 | -121.150 | 2011 | M | Y |
| OmyKR11-WHC19-F1 | Anadromous | White Creek | 46.013 | -121.150 | 2011 | F | N |
| OmyKR11-WHC19-F10 | Anadromous | White Creek | 46.013 | -121.150 | 2011 | F | N |
| OmyKR11-WHC19-G3 | Resident | White Creek | 46.013 | -121.150 | 2011 | M | Y |
| OmyKR11-WHC19-H3 | Anadromous | White Creek | 46.013 | -121.150 | 2011 | F | N |
| OmyKR11-WHC25-B5 | Anadromous | White Creek | 46.013 | -121.150 | 2011 | M | Y |
| OmyKR11-WHC27-A4 | Resident | White Creek | 46.013 | -121.150 | 2011 | F | N |
| OmyKR11-WHC27-B2 | Resident | White Creek | 46.013 | -121.150 | 2011 | M | Y |
| OmyKR11-WHC27-B3 | Resident | White Creek | 46.013 | -121.150 | 2011 | F | N |
| OmyKR11-WHC28-B7 | Resident | White Creek | 46.013 | -121.150 | 2011 | F | N |
| OmyKR11-WHC29-F6 | Resident | White Creek | 46.013 | -121.150 | 2011 | M | Y |
| OmyKR11-WHC30-I3 | Resident | White Creek | 46.013 | -121.150 | 2011 | M | Y |
| OmyKR11-WHC30-J6 | Resident | White Creek | 46.013 | -121.150 | 2011 | M | Y |
| OmyKR11-WHC31-C9 | Anadromous | White Creek | 46.013 | -121.150 | 2011 | F | N |
| OmyKR11-WHC31-J10 | Anadromous | White Creek | 46.013 | -121.150 | 2011 | F | N |
| OmyKR11-WHC32-A5 | Anadromous | White Creek | 46.013 | -121.150 | 2011 | M | Y |
| OmyKR11-WHC32-B3 | Anadromous | White Creek | 46.013 | -121.150 | 2011 | F | N |
| OmyKR11-WHC32-D3 | Anadromous | White Creek | 46.013 | -121.150 | 2011 | F | N |
| OMyKR11-WHC33-H9 | Anadromous | White Creek | 46.013 | -121.150 | 2011 | M | Y |
| OmyKR11-WHC34-I6 | Resident | White Creek | 46.013 | -121.150 | 2011 | M | Y |
| OmyKR11-WHC34-J4 | Anadromous | White Creek | 46.013 | -121.150 | 2011 | F | N |
| OmyKR11-WHC35-A4 | Resident | White Creek | 46.013 | -121.150 | 2011 | M | Y |
| OmyKR11-WHC40-B3 | Resident | White Creek | 46.013 | -121.150 | 2011 | M | Y |
| OmyKR11-WWC14-C2 | Resident | WF White Creek | 46.180 | -121.070 | 2011 | M | Y |
| OmyKR12-BLC12-A8 | Anadromous | Blue Creek | 46.076 | -121.055 | 2012 | F | N |
| OmyKR12-BRC02-D5 | Resident | Brush Creek | 46.163 | -120.945 | 2012 | M | Y |
| OmyKR12-BRC02-G1 | Resident | Brush Creek | 46.163 | -120.945 | 2012 | M | Y |
| OmyKR12-BRC02-G2 | Resident | Brush Creek | 46.163 | -120.945 | 2012 | M | Y |
| OmyKR12-BRC02-H7 | Resident | Brush Creek | 46.163 | -120.945 | 2012 | M | Y |
| OmyKR12-BRC03-E7 | Resident | Brush Creek | 46.163 | -120.945 | 2012 | M | Y |
| OmyKR12-BRC26-C9 | Anadromous | Brush Creek | 46.163 | -120.945 | 2012 | F | N |
| OmyKR12-TPC15-E7 | Anadromous | Tepee Creek | 46.181 | -121.025 | 2012 | F | N |
| OmyKR12-TPC15-J3 | Resident | Tepee Creek | 46.181 | -121.025 | 2012 | M | Y |
| OmyKR12-TPC40-A10 | Anadromous | Tepee Creek | 46.181 | -121.025 | 2012 | M | Y |
| OmyKR12-WHC13-G5 | Resident | White Creek | 46.013 | -121.150 | 2012 | M | Y |
| OmyKR12-WHC22-B10 | Anadromous | White Creek | 46.013 | -121.150 | 2012 | F | N |
| OmyKR12-WHC24-J8 | Resident | White Creek | 46.013 | -121.150 | 2012 | M | Y |
| OmyKR12-WHC31-G7 | Anadromous | White Creek | 46.013 | -121.150 | 2012 | F | N |
| OmyKR12-WHC41-A6 | Anadromous | White Creek | 46.013 | -121.150 | 2012 | F | N |
| OmyKR13-BLC29-D10 | Resident | Blue Creek | 46.076 | -121.055 | 2013 | M | Y |
| OmyKR13-BLC29-G7 | Anadromous | Blue Creek | 46.076 | -121.055 | 2013 | F | N |
| OmyKR13-BRC32-C6 | Anadromous | Brush Creek | 46.163 | -120.945 | 2013 | F | N |
| OmyKR13-BRC33-B1 | Anadromous | Brush Creek | 46.163 | -120.945 | 2013 | U | N |
| OmyKr13-TPC05-A5 | Anadromous | Tepee Creek | 46.181 | -121.025 | 2013 | F | N |
| OmyKR13-TPC08-D9 | Anadromous | Tepee Creek | 46.181 | -121.025 | 2013 | M | Y |
| OmyKR13-TPC10-F6 | Resident | Tepee Creek | 46.181 | -121.025 | 2013 | M | Y |
| OmyKR13-TPC26-A2 | Resident | Tepee Creek | 46.181 | -121.025 | 2013 | M | Y |
| OmyKR13-TPC27-A1 | Resident | Tepee Creek | 46.181 | -121.025 | 2013 | M | Y |
| OmyKR13-WHC13-G5 | Anadromous | White Creek | 46.013 | -121.150 | 2013 | M | Y |
| OmyKR13-WHC14-D7 | Resident | White Creek | 46.013 | -121.150 | 2013 | M | Y |
| OmyKR13-WHC17-A9 | Anadromous | White Creek | 46.013 | -121.150 | 2013 | F | N |
| OmyKR13-WHC19-C2 | Anadromous | White Creek | 46.013 | -121.150 | 2013 | F | N |
| OmyKR13-WHC24-J5 | Anadromous | White Creek | 46.013 | -121.150 | 2013 | F | N |
| OmyKR13-WHC35-E5 | Anadromous | White Creek | 46.013 | -121.150 | 2013 | F | N |
| OmyKR13-WHC35-G4 | Anadromous | White Creek | 46.013 | -121.150 | 2013 | F | N |
| OmyKR13-WHC35-I3 | Anadromous | White Creek | 46.013 | -121.150 | 2013 | M | Y |
| OmyKR13-WHC37-A2 | Anadromous | White Creek | 46.013 | -121.150 | 2013 | F | N |
| OmyKR13-WHC37-H1 | Anadromous | White Creek | 46.013 | -121.150 | 2013 | F | N |
| OmyKR14-BRC12-F7 | Anadromous | Brush Creek | 46.163 | -120.945 | 2014 | F | N |
| OmyKR14-BRC12-G8 | Anadromous | Brush Creek | 46.163 | -120.945 | 2014 | F | N |
| OmyKR14-BRC33-C3 | Anadromous | Brush Creek | 46.163 | -120.945 | 2014 | M | Y |
| OmyKR14-ETC20-C6 | Anadromous | EF Tepee Creek | 46.155 | -121.020 | 2014 | F | N |
| OmyKR14-ETC20-C7 | Anadromous | EF Tepee Creek | 46.155 | -121.020 | 2014 | M | Y |
| OmyKR14-TPC14-A8 | Resident | Tepee Creek | 46.181 | -121.025 | 2014 | M | Y |
| OmyKR14-TPC14-G6 | Anadromous | Tepee Creek | 46.181 | -121.025 | 2014 | F | N |
| OmyKR14-TPC15-A3 | Resident | Tepee Creek | 46.181 | -121.025 | 2014 | M | Y |
| OmyKR14-TPC15-A9 | Resident | Tepee Creek | 46.181 | -121.025 | 2014 | M | Y |
| OmyKR14-TPC24-A2 | Anadromous | Tepee Creek | 46.181 | -121.025 | 2014 | M | Y |
| OmyKR14-TPC25-A3 | Anadromous | Tepee Creek | 46.181 | -121.025 | 2014 | M | Y |
| OmyKR14-TPC25-D3 | Anadromous | Tepee Creek | 46.181 | -121.025 | 2014 | F | N |
| OmyKR14-WHC34-A3 | Resident | White Creek | 46.013 | -121.150 | 2014 | M | Y |
| OmyKR14-WHC34-B2 | Anadromous | White Creek | 46.013 | -121.150 | 2014 | F | N |
| OmyKR14-WHC34-D7 | Anadromous | White Creek | 46.013 | -121.150 | 2014 | F | N |
| OmyKR14-WHC35-G2 | Resident | White Creek | 46.013 | -121.150 | 2014 | M | Y |
| OmyKR14-WHC35-J2 | Anadromous | White Creek | 46.013 | -121.150 | 2014 | M | Y |
| OmyKR14-WHC35-J9 | Anadromous | White Creek | 46.013 | -121.150 | 2014 | M | Y |
| OmyKR14-WHC37-G8 | Resident | White Creek | 46.013 | -121.150 | 2014 | M | Y |
| OmyKR14-WHC38-C3 | Anadromous | White Creek | 46.013 | -121.150 | 2014 | F | N |
| OmyKR14-WHC39-G3 | Anadromous | White Creek | 46.013 | -121.150 | 2014 | M | Y |
| OmyKR15-BRC20-A2 | Anadromous | Brush Creek | 46.163 | -120.945 | 2015 | F | N |
| OmyKR15-TPC21-C10 | Anadromous | Tepee Creek | 46.181 | -121.025 | 2015 | M | Y |
| OmyKR15-TPC25-G6 | Anadromous | Tepee Creek | 46.181 | -121.025 | 2015 | M | Y |
| OmyKR15-TPC25-G9 | Anadromous | Tepee Creek | 46.181 | -121.025 | 2015 | F | N |
| OmyKR15-TPC25-I1 | Anadromous | Tepee Creek | 46.181 | -121.025 | 2015 | F | N |
| OmyKR15-WHC20-C1 | Resident | White Creek | 46.013 | -121.150 | 2015 | M | Y |
| OmyKR15-WHC29-I7 | Anadromous | White Creek | 46.013 | -121.150 | 2015 | M | Y |
| OmyKR15-WHC31-A9 | Resident | White Creek | 46.013 | -121.150 | 2015 | M | Y |
| OmyKR16-BRC12-B7 | Resident | Brush Creek | 46.163 | -120.945 | 2016 | M | Y |
| OmyKR16-BRC12-E2 | Anadromous | Brush Creek | 46.163 | -120.945 | 2016 | F | N |
| OmyKR16-BRC20-C4 | Anadromous | Brush Creek | 46.163 | -120.945 | 2016 | F | N |
| OmyKR16-BRC28-D3 | Resident | Brush Creek | 46.163 | -120.945 | 2016 | M | Y |
| OmyKR16-TPC15-A2 | Resident | Tepee Creek | 46.181 | -121.025 | 2016 | M | Y |
| OmyKR16-TPC15-A4 | Resident | Tepee Creek | 46.181 | -121.025 | 2016 | M | Y |
| OmyKR16-TPC36-A5 | Anadromous | Tepee Creek | 46.181 | -121.025 | 2016 | F | N |
| OmyKR16-WHC19-C4 | Anadromous | White Creek | 46.013 | -121.150 | 2016 | F | N |
| OmyKR16-WHC19-C5 | Anadromous | White Creek | 46.013 | -121.150 | 2016 | F | N |
| OmyKR16-WHC23-A7 | Anadromous | White Creek | 46.013 | -121.150 | 2016 | F | N |
| OmyKR16-WHC23-D7 | Anadromous | White Creek | 46.013 | -121.150 | 2016 | M | Y |
| OmyKR16-WHC23-D8 | Anadromous | White Creek | 46.013 | -121.150 | 2016 | M | Y |
| OmyKR16-WHC23-G2 | Anadromous | White Creek | 46.013 | -121.150 | 2016 | M | Y |
| OmyKR16-WHC23-H5 | Resident | White Creek | 46.013 | -121.150 | 2016 | M | Y |
| OmyKR16-WHC23-I4 | Resident | White Creek | 46.013 | -121.150 | 2016 | M | Y |
| OmyKR16-WHC23-I9 | Anadromous | White Creek | 46.013 | -121.150 | 2016 | F | N |
| OmyKR16-WHC25-A5 | Anadromous | White Creek | 46.013 | -121.150 | 2016 | F | N |
| OmyKR16-WHC25-C3 | Anadromous | White Creek | 46.013 | -121.150 | 2016 | F | N |
| OmyKR16-WHC25-C5 | Anadromous | White Creek | 46.013 | -121.150 | 2016 | M | Y |
| OmyKR16-WHC26-A6 | Resident | White Creek | 46.013 | -121.150 | 2016 | M | Y |
| OmyKR16-WHC27-A7 | Resident | White Creek | 46.013 | -121.150 | 2016 | M | Y |
| OmyKR16-WHC27-F3 | Resident | White Creek | 46.013 | -121.150 | 2016 | M | Y |
| OmyKR16-WHC27-G8 | Resident | White Creek | 46.013 | -121.150 | 2016 | M | Y |
| OmyKR16-WHC29-C1 | Anadromous | White Creek | 46.013 | -121.150 | 2016 | F | N |
| OmyKR17-BRC02-B2 | Resident | Brush Creek | 46.163 | -120.945 | 2017 | M | N |
| OmyKR17-BRC02-B7 | Resident | Brush Creek | 46.163 | -120.945 | 2017 | M | Y |
| OmyKR17-BRC02-D6 | Resident | Brush Creek | 46.163 | -120.945 | 2017 | M | Y |
| OmyKR17-BRC04-B8 | Resident | Brush Creek | 46.163 | -120.945 | 2017 | M | Y |
| OmyKR17-BRC04-D6 | Resident | Brush Creek | 46.163 | -120.945 | 2017 | M | Y |
| OmyKR17-BRC04-E5 | Resident | Brush Creek | 46.163 | -120.945 | 2017 | M | Y |
| OmyKR17-TPC03-F6 | Resident | Tepee Creek | 46.181 | -121.025 | 2017 | M | Y |
| OmyKR17-TPC07-A9 | Resident | Tepee Creek | 46.181 | -121.025 | 2017 | M | Y |
| OmyKR17-TPC07-F10 | Resident | Tepee Creek | 46.181 | -121.025 | 2017 | M | Y |
| OmyKR17-WHC06-C8 | Anadromous | White Creek | 46.013 | -121.150 | 2017 | F | N |
| OmyKR17-WHC07-H3 | Resident | White Creek | 46.013 | -121.150 | 2017 | M | Y |
| OmyKR17-WHC07-H5 | Resident | White Creek | 46.013 | -121.150 | 2017 | M | Y |
| OmyKR17-WHC08-A6 | Resident | White Creek | 46.013 | -121.150 | 2017 | M | Y |
| OmyKR17-WHC08-F4 | Resident | White Creek | 46.013 | -121.150 | 2017 | M | Y |
| OmyKR17-WHC10-A10 | Resident | White Creek | 46.013 | -121.150 | 2017 | M | Y |
| OmyKR17-WHC10-E6 | Resident | White Creek | 46.013 | -121.150 | 2017 | M | Y |
| OmyKR18-BRC01-C8 | Anadromous | Brush Creek | 46.163 | -120.945 | 2018 | M | Y |
| OmyKR18-BRC02-D9 | Anadromous | Brush Creek | 46.163 | -120.945 | 2018 | F | N |
| OmyKR18-BRC04-B5 | Anadromous | Brush Creek | 46.163 | -120.945 | 2018 | F | N |
| OmyKR18-BRC04-B6 | Anadromous | Brush Creek | 46.163 | -120.945 | 2018 | F | N |
| OmyKR18-BRC04-G5 | Anadromous | Brush Creek | 46.163 | -120.945 | 2018 | F | N |
| OmyKR18-BRC05-A4 | Anadromous | Brush Creek | 46.163 | -120.945 | 2018 | M | Y |
| OmyKR18-BRC05-B6 | Anadromous | Brush Creek | 46.163 | -120.945 | 2018 | F | N |
| OmyKR18-BRC05-D1 | Anadromous | Brush Creek | 46.163 | -120.945 | 2018 | F | N |
| OmyKR18-TPC01-A10 | Anadromous | Tepee Creek | 46.181 | -121.025 | 2018 | F | N |
| OmyKR18-TPC02-C5 | Anadromous | Tepee Creek | 46.181 | -121.025 | 2018 | F | N |
| OmyKR18-TPC02-D4 | Anadromous | Tepee Creek | 46.181 | -121.025 | 2018 | M | Y |
| OmyKR18-TPC02-E3 | Anadromous | Tepee Creek | 46.181 | -121.025 | 2018 | M | Y |
| OmyKR18-TPC02-G6 | Anadromous | Tepee Creek | 46.181 | -121.025 | 2018 | F | N |
| OmyKR18-TPC03-C2 | Anadromous | Tepee Creek | 46.181 | -121.025 | 2018 | F | N |
| OmyKR18-TPC03-F2 | Anadromous | Tepee Creek | 46.181 | -121.025 | 2018 | F | N |
| OmyKR18-TPC05-A9 | Anadromous | Tepee Creek | 46.181 | -121.025 | 2018 | F | N |
| OmyKR18-TPC05-B4 | Anadromous | Tepee Creek | 46.181 | -121.025 | 2018 | F | N |
| OmyKR18-TPC05-I9 | Anadromous | Tepee Creek | 46.181 | -121.025 | 2018 | M | Y |
| OmyKR18-TPC05-J6 | Anadromous | Tepee Creek | 46.181 | -121.025 | 2018 | F | N |
| OmyKR18-TPC06-A1 | Anadromous | Tepee Creek | 46.181 | -121.025 | 2018 | M | N |
| OmyKR18-TPC06-A8 | Anadromous | Tepee Creek | 46.181 | -121.025 | 2018 | F | N |
| OmyKR18-TPC06-C8 | Anadromous | Tepee Creek | 46.181 | -121.025 | 2018 | M | Y |
| OmyKR18-TPC11-B4 | Anadromous | Tepee Creek | 46.181 | -121.025 | 2018 | F | N |
| OmyKR18-WHC04-E6 | Anadromous | White Creek | 46.013 | -121.150 | 2018 | F | N |
| OmyKR18-WHC06-H8 | Anadromous | White Creek | 46.013 | -121.150 | 2018 | F | N |
| OmyKR18-WHC08-B9 | Anadromous | White Creek | 46.013 | -121.150 | 2018 | M | Y |
| OmyKR18-WHC16-C9 | Anadromous | White Creek | 46.013 | -121.150 | 2018 | M | Y |
| OmyKR18-WHC19-D8 | Anadromous | White Creek | 46.013 | -121.150 | 2018 | F | N |
| OmyKR18-WHC19-F8 | Anadromous | White Creek | 46.013 | -121.150 | 2018 | M | Y |
| OmyKR18-WHC19-H9 | Anadromous | White Creek | 46.013 | -121.150 | 2018 | F | N |
| OmyKR18-WHC20-A7 | Anadromous | White Creek | 46.013 | -121.150 | 2018 | M | Y |
| OmyKR18-WHC20-G8 | Anadromous | White Creek | 46.013 | -121.150 | 2018 | M | Y |
| OmyKR19-WHC09-J6 | Anadromous | White Creek | 46.013 | -121.150 | 2019 | M | Y |
