# Supplementary material for: Whole-Genome Resequencing to Evaluate Life History Variation in Anadromous Migration of Oncorhynchus mykiss
Source: Front Genet. 2022 Mar 15;13:795850. doi: 10.3389/fgene.2022.795850 (PMC8964970; doi:10.3389/fgene.2022.795850)
Supplement: Supplementary file 7 [file DataSheet2.docx]

**Supplementary Table 1.** Sample names, life history types, sample tributary, and years.

**Supplementary Figure 1.** Local score Manhattan plot. The minimum coverage threshold was 15 reads and the minor allele frequency was ≥ 0.05.

**Supplementary Figure 2.** Manhattan plot of significance of local scores. The minimum coverage threshold was 15 reads and the minor allele frequency was ≥ 0.05.

**Supplementary Figure 3.** Manhattan plot of F_ST_ scores. The minimum coverage threshold was 15 reads and the minor allele frequency was ≥ 0.05.

**Supplementary Figure 4**. Number of overlapping loci and correlation of loci at different numbers of loci retained in Random Forest (RF) analysis.

**Supplementary Figure 5.** Local score Manhattan plot with male and female *O. mykiss* individuals included. The minimum coverage threshold was 15 reads and minor allele frequency was *≥* 0.05.

**Supplementary Figure 6.** QQ-plot of observed compared to expected sliding window F_ST_ (sF_ST_) data for all loci analyzed. The red line represents the threshold of significant sF_ST_ values and all loci surpassing the significance threshold are labelled.
